# Specific polyunsaturated fatty acids modulate lipid delivery and oocyte development in *C. elegans* revealed by molecular-selective label-free imaging

**DOI:** 10.1038/srep32021

**Published:** 2016-08-18

**Authors:** Wei-Wen Chen, Yung-Hsiang Yi, Cheng-Hao Chien, Kuei-Ching Hsiung, Tian-Hsiang Ma, Yi-Chun Lin, Szecheng J. Lo, Ta-Chau Chang

**Affiliations:** 1 Institute of Atomic and Molecular Sciences, Academia Sinica, Taipei 106, Taiwan; 2 Molecular Science and Technology Program, Taiwan International Graduate Program, Academia Sinica, Taipei 106, Taiwan; 3 Department of Chemistry, National Tsing Hua University, Hsinchu 300, Taiwan; 4 Center of Molecular Medicine, College of Medicine, Chang Gung University, Kwei-Shan, Tao-Yuan 333, Taiwan; 5 Department of Biomedical Sciences, College of Medicine, Chang Gung University, Kwei-Shan, Tao-Yuan 333, Taiwan

## Abstract

Polyunsaturated fatty acids (PUFAs) exhibit critical functions in biological systems and their importance during animal oocyte maturation has been increasingly recognized. However, the detailed mechanism of lipid transportation for oocyte development remains largely unknown. In this study, the transportation of yolk lipoprotein (lipid carrier) and the rate of lipid delivery into oocytes in live *C. elegans* were examined for the first time by using coherent anti-Stokes Raman scattering (CARS) microscopy. The accumulation of secreted yolk lipoprotein in the pseudocoelom of live *C. elegans* can be detected by CARS microscopy at both protein (~1665 cm^−1^) and lipid (~2845 cm^−1^) Raman bands. In addition, an image analysis protocol was established to quantitatively measure the levels of secreted yolk lipoprotein aberrantly accumulated in PUFA-deficient *fat* mutants (*fat-1*, *fat-2*, *fat-3*, *fat-4*) and PUFA-supplemented *fat-2* worms (the PUFA add-back experiments). Our results revealed that the omega-6 PUFAs, not omega-3 PUFAs, play a critical role in modulating lipid/yolk level in the oocytes and regulating reproductive efficiency of *C. elegans*. This work demonstrates the value of using CARS microscopy as a molecular-selective label-free imaging technique for the study of PUFA regulation and oocyte development in *C. elegans*.

The successful development of oocytes and embryos is one of the most important events for species survival, in which unsaturated fatty acids act as fuel molecules, the building blocks of membranes, and signaling molecules in various pathways^1^. The biosynthesis of unsaturated fatty acids including polyunsaturated fatty acids (PUFAs) follows a conserved mechanism from bacteria to human^2^. Therefore, one can uncover the regulation of PUFAs by using different animal models. Similar results from mice and *C. elegans* suggest that sufficient oocyte PUFAs are necessary for fertilization because these PUFAs are precursors of the prostaglandins that control sperm-oocyte interactions^3^,^4^. Since *C. elegans* has a well-defined anatomy, short life cycle, and genetic similarity to the human genome, it has been widely used for studies of the relationship between lipid/PUFAs and the signaling pathways of reproductive growth^5^, development^6^, and reproduction^7^,^8^. Moreover, because of its optically transparent body, *C. elegans* is considered an excellent animal model for optical studies, such as high-speed super-resolution microscopy^9^, optogenetic manipulation^10^, and non-linear optical techniques^11^.

Yolk lipoprotein is the major carrier that transfers lipids/fatty acids from the intestine to oocytes in *C. elegans*^12^. It is believed that a continuum exists between nascent lipid droplets (LDs) and immature yolk particles during the formation of yolk lipoprotein, which is analogous to the production of lipoprotein in mammals^13^. However, the underlying mechanism of the incorporation of yolk lipoproteins/LDs remains unknown. The protein motif of yolk lipoprotein, vitellogenins, is homologous to human apoB^14^,^15^. The endocytosis of yolk lipoprotein in oocytes of *C. elegans* is mediated by a specific receptor, RME-2 (encoded by *rme-2* gene), in a similar fashion as human apoB^16^. Lipid metabolism in oocytes is critical for the growth of oocytes, fertilization, and the development of early embryos^1^. Failure of proper lipid delivery leads to abnormal oocytes, a low egg production rate, and low viability of embryos^16^,^17^.

*C. elegans* can synthesize *de novo* a wide range of PUFAs by converting the oleic acid (18:1,n9) by a series of desaturases and elongases^18^. Figure S1a shows the biosynthesis of PUFAs and the genes involved in the desaturation: *fat-1*, *fat-2*, *fat-3*, and *fat-4*. Deficiency in PUFAs, due to the dysfunction of the desaturases, can cause defects in lipid regulation and reproduction. For example, the *fat-2* mutant (lack of all PUFAs) decreases in size, number, content of LDs dramatically^19^, and has a significantly low brood size (~19%) and hatching rate (29%) compared to wild-type worms^17^. PUFAs have been characterized as the precursor of signaling factors that control the recruitment of sperm^3^,^20^,^21^ and are essential materials for synthesizing the lipid-rich layer of eggshells during oocyte maturation^22^. These findings suggested that PUFAs are important for oocyte development and reproduction. Although the process of oocyte development has been studied^23^,^24^, the detailed mechanism of how PUFAs modulate oocyte development remains unclear.

Since adult hermaphrodites turnover their entire gonad every 6.5 hours^25^, the cellular events, including oocyte growth, maturation, and ovulation, are highly coordinated to ensure successful fertilization^23^,^24^. The presence of lipids is necessary for the development of oocytes and embryos because it is a highly energy-consuming process^26^,^27^. Although some signaling factors such as maturation-promoting factor^28^ and major sperm protein^29^ have been characterized to regulate the development of oocytes, there is no *in vivo* evidence for delineating how PUFAs regulate oocyte development. A number of methods have been developed and applied to analyze lipid content in oocytes, including dye staining^30^, isotopic labeling^31^, gas chromatography (GC) and matrix assisted laser desorption/ ionization mass spectrometry (MALDI-MS)^32^. However, these methods need fixation, labeling, dissection, or purification. On the other hand, coherent Raman techniques have several advantages including non-invasiveness, high molecular specificity, and high image quality, which makes them powerful tools for characterization and quantification of the molecular dynamics in cells, tissues, and animal models^33^,^34^,^35^,^36^. Coherent Raman techniques, such as coherent anti-Stokes Raman scattering (CARS), have been applied to lipid studies in *C. elegans* since 2007^37^; however, no studies on oocyte development have been reported.

CARS microscopy provides a means of imaging the reproductive system in *C. elegans* including germ line, oocytes, spermatheca, and embryos (Fig. S1b). In the present study, the *fat* mutants (*fat*-1~*fat*-4) were introduced as exemplars of animals, where yolk uptake is defective, to investigate which kind of PUFA (omega-3 or omega-6) is more critical to the transportation of yolk lipoprotein into oocytes. We showed that CARS microscopy is capable of detecting the aberrant accumulation of secreted yolk lipoprotein in the pseudocoelomic cavity without any labeling by using protein (~1665 cm^−1^) and lipid (~2845 cm^−1^) Raman bands. In addition, we established an image analysis protocol to identify and quantitatively measure the levels of secreted yolk lipoprotein accumulation in *fat* mutants and PUFA-supplemented *fat-2* worms (the PUFA add-back experiments). Finally, we examined the lipid content in single oocytes, the development of the oocyte (including oocyte size, ovulation rate, and egg number), and quantitatively estimated the rate of lipid delivery into oocytes in *C. elegans*. Our data suggest that omega-6 PUFAs are a key regulator for lipid delivery and oocyte development in *C. elegans*.

## Results

### Detection of secreted yolk lipoprotein accumulated in the pseudocoelom of *C. elegans* by CARS imaging

**Figure 1:**
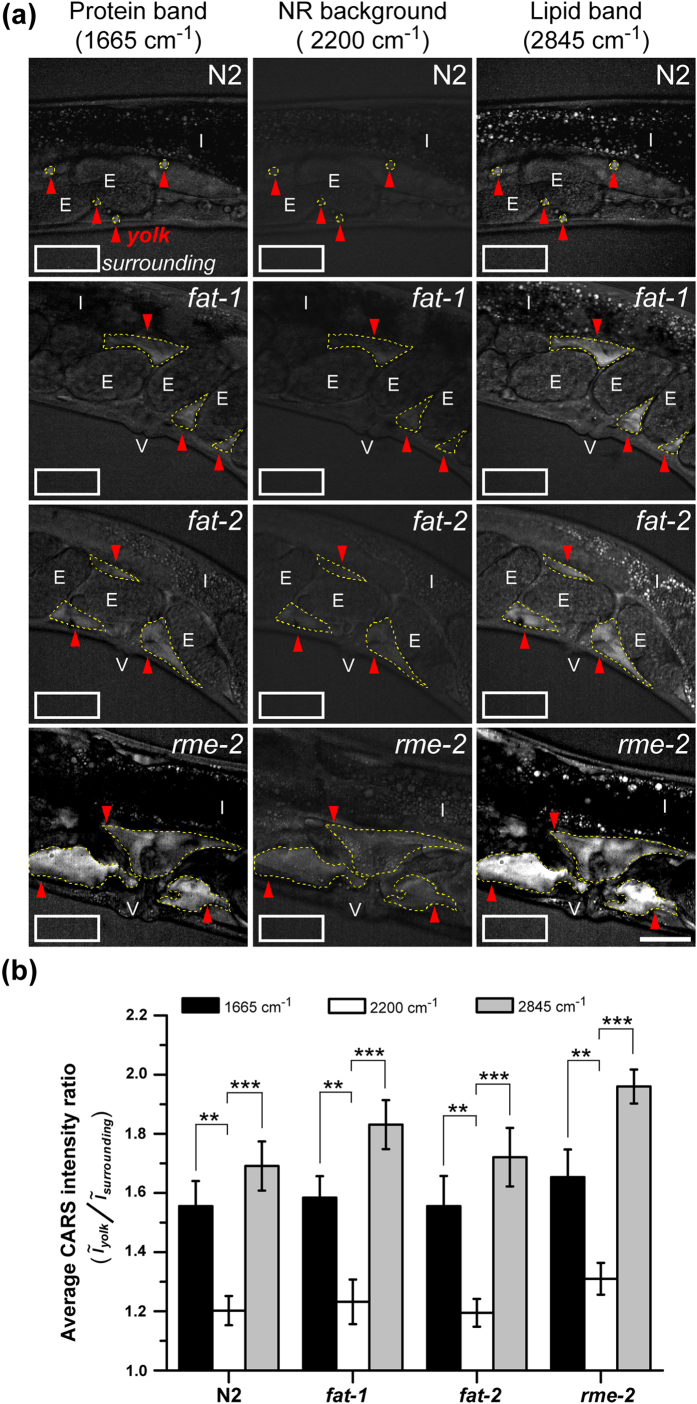
The accumulation of secreted yolk lipoprotein in the pseudocoelom of N2, *fat-1*, *fat-2*, and *rme-2* mutants. (**a**) CARS Images of N2, *fat-1*, *fat-2*, and *rme-2* mutants containing yolk lipoprotein accumulation measured at three different vibrational frequencies. Red arrows and white rectangles represent the regions of yolk lipoprotein accumulation and surrounding buffer, respectively. E stands for embryo, I stands for intestine, and V stands for vulva. Scale bar = 30 μm. (**b**) The average CARS intensity ratio between yolk lipoprotein accumulation (red arrows in (**a**)) and surrounding buffer (white rectangles in (**a**)) at three vibrational frequencies. Black bar for ~1665 cm^−1^ (protein band), white bar for ~2200 cm^−1^ (non-resonant background), and gray bar for ~2845 cm^−1^ (lipid band) (n = 10~15). All worms are 1D-Ad worms. Error bars represent the standard error of the mean (SEM). (P-value: *p < 0.05, **p < 0.01, ***p < 0.001).

In addition to the use of the lipid Raman band at ~2845 cm^−1^ (CH_2_ stretching) for monitoring the accumulation of the secreted yolk lipoprotein in the pseudocoelomic cavity of uterus^19^, the protein Raman band at ~1665 cm^−1^ (amide I/C=C stretching)^38^ provides an additional marker for CARS imaging to identify the yolk lipoprotein accumulation. We first examined the accumulation of secreted yolk lipoprotein in one-day-adult (1D-Ad) *rme-2*, *fat-1*, and *fat-2* mutants by Raman micro-spectroscopy. The Raman data confirm that 1665 cm^−1^ is a reliable protein Raman band (Fig. S2)^38^. We then used these two Raman bands at ~1665 cm^−1^ (protein band) and ~2845 cm^−1^ (lipid band) plus the non-resonant background at ~2200 cm^−1^ to image the aberrant accumulation of secreted yolk lipoprotein in these mutants and wild-type N2 worms (Fig. 1a). The CARS images show high co-localization between both Raman bands at ~1665 cm^−1^ and ~2845 cm^−1^ (Fig. S3). The average CARS intensity ratio between secreted yolk lipoprotein accumulations and surrounding buffer suggests that both the protein band ratio and lipid band ratio are significantly higher than the ratio at non-resonant background in all four *C. elegans* strains (Fig. 1b). These indicate that both CARS signals of protein and lipid bands provide a sufficient S/N ratio for *in vivo* imaging of secreted yolk lipoprotein accumulated in *C. elegans*.

We further used this method to measure the distribution of the protein/lipid ratio for lipid-enriched particles in the N2 intestine (data not shown). However, we were not able to precisely distinguish yolk particles from LDs in the intestine. This is because there are various yolk/LD intermediates during yolk biosynthesis and there are no specific protein or lipid markers that can definitively distinguish intestinal yolk and LDs at present^13^. Because CARS imaging has a higher S/N ratio of secreted yolk lipoprotein accumulation at lipid band (~2845 cm^−1^) than at protein band (~1665 cm^−1^), the CARS imaging of secreted yolk lipoprotein at ~2845 cm^−1^ was used for data collection and analysis for the following experiments.

### Analysis of secreted yolk lipoprotein accumulation in the pseudocoelom of worms

**Figure 2:**
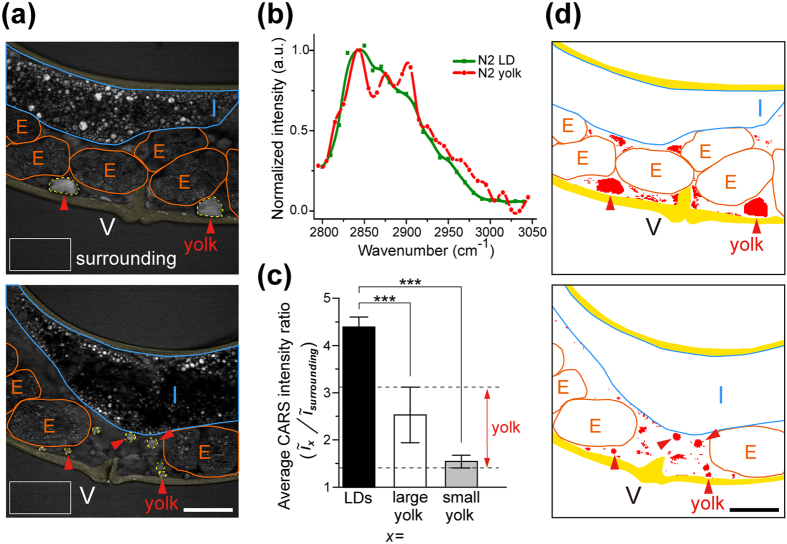
Analysis of yolk lipoprotein accumulation in the pseudocoelomic cavity of one-day-adult (1D-Ad) wild-type (N2) worms. (**a**) The CARS image of 1D-Ad N2 worms containing large (upper image) and small (lower image) yolk lipoprotein accumulation (red arrows) in their pseudocoelomic cavities. The white rectangles are selected for calculating the average CARS intensity of surrounding buffer. Yellow region stands for the skin-like hypodermal cells, E stands for embryo, I stands for intestine, and V stands for vulva. Scale bar = 30 μm. (**b**) The CARS spectra of yolk lipoprotein accumulation obtained from pseudocoelomic cavity and the lipid droplet (LD) obtained from the skin-like hypodermal cell in 1D-Ad N2 worms. (**c**) The average CARS intensity ratios between LDs/surrounding buffer and between (large and small) yolk lipoprotein accumulation/surrounding buffer. A threshold of 1.47–3.12 (in red color) was applied to the CARS image to enhance the boundary of yolk lipoprotein accumulation. Error bars represent the standard deviation. (P-value: *p < 0.05, **p < 0.01, ***p < 0.001) (**d**) After excluding the internal organs/tissues (such as intestine, oocyte/embryo, and body wall) and applying the threshold, the pixels with intensity ratio between 1.47–3.12 are converted into red color, and pixels with intensity ratio <1.47 and >3.12 are converted into white color. The boundary of yolk lipoprotein accumulation can be easily distinguished (indicated by red arrows).

Here we examined the CARS signals at ~2845 cm^−1^ of the secreted yolk lipoprotein accumulations in the pseudocoelom (Fig. 2a) and the LDs in the skin-like hypodermal cells (Fig. S4). We found that both of them show strong lipid signal in CARS imaging (Fig. 2a and Fig. S4) and in normalized CARS spectrum (Fig. 2b). In contrast to LDs, the secreted yolk lipoprotein accumulation in pseudocoelom is larger in size with polygonal shapes and has weaker average CARS intensity. The average CARS intensity ratio between secreted yolk lipoprotein accumulation and the surrounding buffer (2.53 ± 0.59 and 1.69 ± 0.22 for large and small accumulations, respectively) is much weaker than the ratio between the LDs and surrounding buffer (4.39 ± 0.21) (Fig. 2c) in 1D-Ad wild-type worms. After excluding the internal organs/tissues such as intestine, oocytes/embryos, and body wall, we set a threshold at an intensity ratio within 1.47–3.12 to convert the CARS image into a binary image for extracting the component of secreted yolk lipoprotein accumulation in the pseudocoelomic cavity (as indicated by red arrows in Fig. 2a,d). We further introduced the signal of VIT-2-GFP reporter as a reference to test the detection limit and found that this thresholding method is capable of detecting the accumulation of secreted yolk lipoprotein with size ≥10 μm^2^ (Fig. S5). Accordingly, the size histogram of all the accumulations of secreted yolk lipoprotein in the pseudocoelomic cavity of uterus in 1D-Ad wild-type worms was plotted (Fig. S6a). After the number of occurrence was normalized by the number of worms (n = 57) (Fig. S6b) and multiplied by each binned area size, the area contribution curve of the wide-type worm can be obtained (Fig. S6c) (also see Methods for the details). The results show that the most dominant size of secreted yolk lipoprotein accumulation is 20~60 μm^2^ and the major contribution to the total area (~72%) of secreted yolk lipoprotein accumulation is <80 μm^2^ for N2 worms. This method was also applied to examine the size distribution of secreted yolk lipoproteins in the pseudocoelomic cavity of *fat* mutants.

### PUFAs are required for the transportation of yolk lipoprotein into oocytes

**Figure 3:**
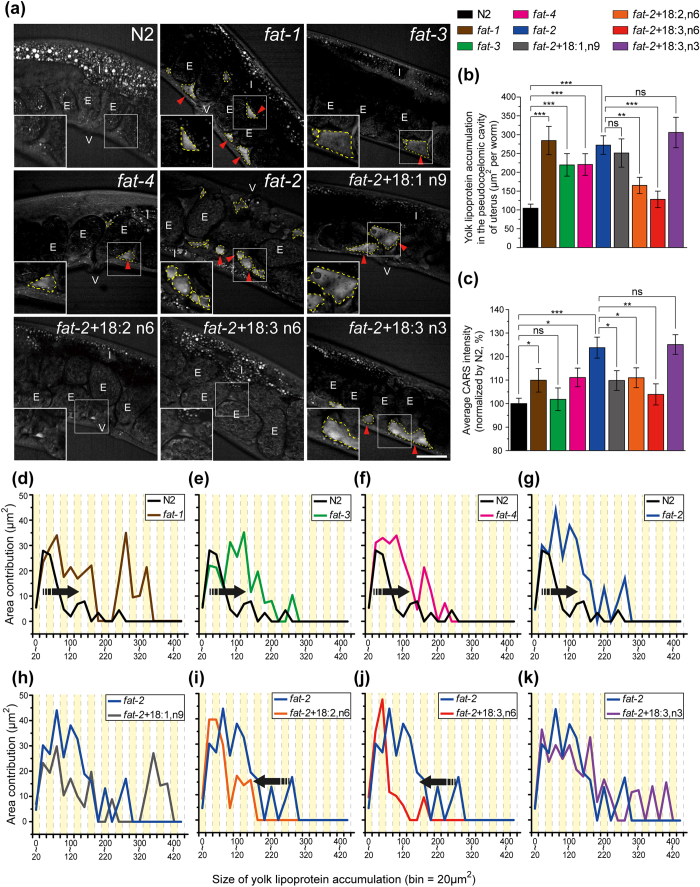
Analysis of yolk lipoprotein accumulation in PUFA-deficient mutants. (**a**) The CARS images of N2, PUFA-deficient mutants (*fat-1*, *fat-2*, *fat-3*, and *fat-4*), and *fat-2* worms supplemented with PUFAs. Red arrows indicate the regions of yolk lipoprotein accumulation. E stands for embryo, I stands for intestine, and V stands for vulva. Scale bar = 30 μm. (**b**) The total area and (**c**) the average CARS intensity of yolk lipoprotein accumulations in the pseudocoelomic cavity of uterus in different worms. (n = 57 for N2, and n = 19~32 for mutants and PUFA-supplemented *fat-2* worms) All worms are 1D-Ad worms. Error bars represent the SEM. (**d–k**) The area contribution curves of yolk lipoprotein accumulations in various strains. (P-value: *p < 0.05, **p < 0.01, ***p < 0.001, ns: not significant).

We next measured the accumulation level of secreted yolk lipoprotein in different mutants (*fat-1*, *fat-2*, *fat-3*, and *fat-4*) and examined the effects of various PUFA supplementations (18:1,n9, 18:2,n6, 18:3,n6, and 18:3,n3 fatty acids) to *fat-2* mutants (Fig. 3a,b). These *fat* mutants were chosen because they exhibit different levels of defects in PUFA biosynthesis. Consequently, their phenotypes could act as markers for investigating the role of PUFAs in the transportation of yolk lipoprotein in *C. elegans*. The supplementation of various PUFAs to *fat-2* worms was an add-back method to study the differences between complete deficiency in PUFAs (*fat-2*) and either partial (18:3,n6 and 18:3,n3) or total (18:2,n6) restoration of PUFA production in the intestine of *fat-2* worms. The supplementation of MUFA (18:1,n9) was applied as a negative control. All the *fat-1* to *fat-4* mutants show generally enhanced CARS intensities (Fig. 3c) and dramatically increased levels of secreted yolk lipoprotein accumulation (Fig. 3b). The major sizes of accumulations in these mutants are all larger than N2 worms (Fig. 3d–g). The supplementation of omega-6 PUFAs in *fat-2* worms exhibits appreciably reduced accumulation level (Fig. 3b), average CARS intensity (Fig. 3c), and the size of secreted yolk lipoprotein accumulation (Fig. 3i,j). However, we did not observe a significant difference after the dietary restoration of MUFA (Fig. 3b,c,h) and omega-3 PUFAs (Fig. 3b,c,k). These data indicate that PUFA is a key factor for the transportation of yolk lipoprotein. To further determine which type of PUFA (omega-6 or omega-3) is more critical for the oocyte development, it is necessary to examine the lipid content in oocytes.

### Quantitative analysis of lipid content in −1 to −4 oocytes during oocyte development

**Figure 4:**
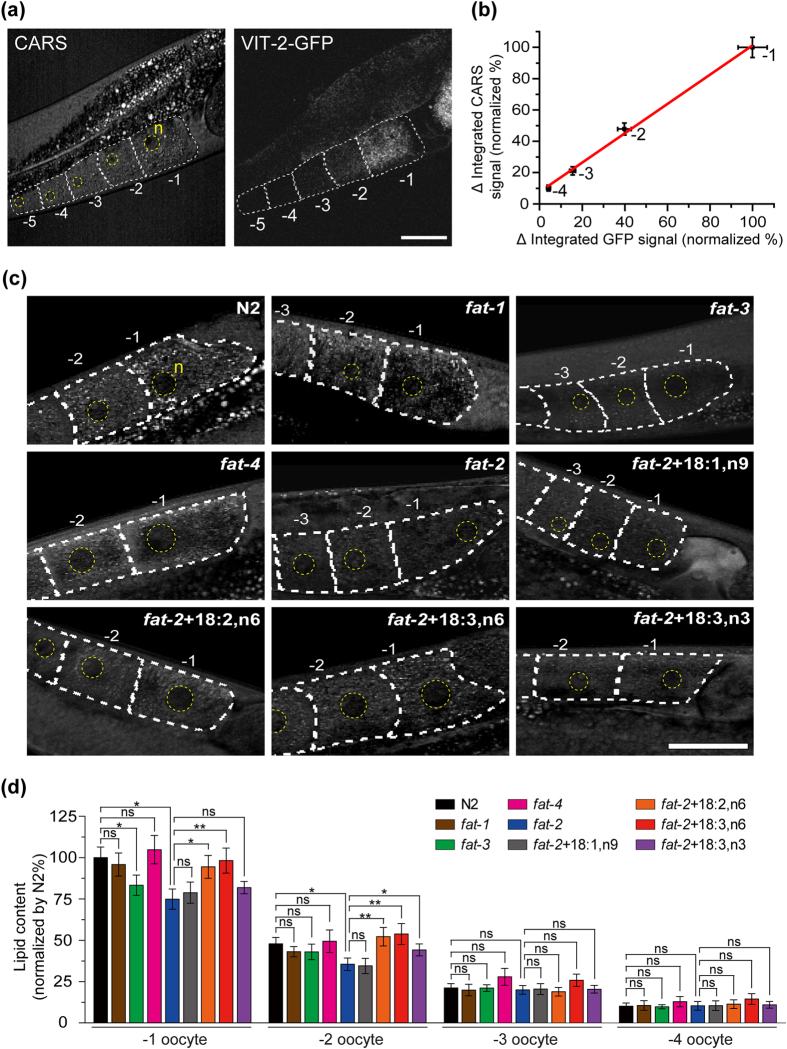
Quantitative analysis of lipid content in oocytes. (**a**) CARS and two-photon excitation fluorescence images of the oocytes of a wild-type worm with *vit-2p::vit-2::gfp* transgene expression. n: nucleus. (**b**) The correlation between Δ integrated GFP signal and Δ integrated CARS signal of each oocyte. The red line represents the linear regression (R^2^ > 0.99). Each data point represents the average ± SEM (n = 17). (**c**) The CARS images of the oocytes and (**d**) The normalized amount of lipid stored in the oocytes of N2, *fat-1*, *fat-3*, *fat-4*, *fat-2*, and *fat-2* worms supplemented with 18:1,n9, 18:2,n6, 18:3,n6, and 18:3,n3 (n = 9~11). All are 1D-Ad worms. Error bars represent the SEM. Scale bar = 30 μm. (P-value: *p < 0.05, **p < 0.01, ***p < 0.001. ns: not significant).

We simultaneously obtained both CARS and two-photon excitation fluorescence images of the oocytes in the wild-type worms with *vit-2p::vit-2::gfp* transgene expression (Fig. 4a) and, then analyzed the integrated CARS and GFP fluorescence signals (see Methods for the definition of integrated CARS/GFP signal) in these images to investigate the lipid delivery into oocytes by yolk lipoprotein during oocyte development in *C. elegans*. The GFP fluorescence signal shows the distribution of the GFP-tagged yolk lipoprotein (VIT-2-GFP), whereas the CARS signal shows the lipid content in oocytes. We noticed that the −1 oocyte has the highest GFP fluorescence signal and decreasing signal in the following oocytes (−2, −3, −4 and −5), indicating that VIT-2-GFP yolk lipoprotein complexes transport into the proximal oocytes that are nearly mature (−1 and −2 oocytes), but not to the immature oocytes such as the −5 oocyte. This is because the GFP signal in the −5 oocyte was almost non-detectable and could be ignored (Fig. 4a). To confirm the linear correlation between lipid carrier (revealed by GFP signal) and lipid stored (revealed by CARS signal) in each oocyte, Fig. 4b shows the plot of Δ integrated CARS signal *versus* Δ integrated GFP signal for each oocyte (−1, −2, −3 and −4). The Δ integrated CARS signal was defined as the difference in integrated CARS signal of each oocyte to that of the −5 oocyte (as an offset), and so was the Δ integrated GFP signal. The strong linear correlation (R^2^ > 0.99) between them indicates that CARS signal can reveal the amount of lipid delivered into oocytes by yolk lipoproteins.

We further examined the oocytes in *fat-1* to *fat-4* mutants, and PUFA-supplemented *fat-2* mutant (Fig. 4c). We found that N2, *fat-1*, and *fat-4* worms show a similar level of lipid content in their oocytes, but *fat-2* and *fat-3* mutants showed a discernible decrease of lipid content in the −2 or −1 oocytes (Fig. 4d). This implies that the delivery of lipid into oocytes is not affected by the deficiency of omega-3 PUFAs in *fat-1* mutants. The *fat-3* mutants, which lose most of their PUFAs due to the dysfunction of delta-6 desaturase, show less lipid content in its −1 oocytes. The loss of both omega-3 and omega-6 PUFAs in *fat-2* compromised the delivery of lipid into oocytes. The average lipid content in both −1 and −2 oocytes of *fat-2* mutant is ~25% less than N2 worms. Supplementation with omega-6 PUFAs to *fat-2* mutant could recover the lipid content in the −1 and −2 oocytes to a level close to that of N2 worms, while supplementation with MUFA (18:1,n9) or omega-3 PUFA (18:3,n3) did not significantly improve the level of lipid storage in oocytes in *fat-2* mutants (p = 0.666 for 18:1,n9 and p = 0.329 for 18:3,n3) (Fig. 4d). These data suggest that omega-6 PUFAs are necessary for the lipid delivery into oocytes in *C. elegans*, which is critical for oocyte development and embryonic development.

### Omega-6 PUFAs modulate oocyte development in *C. elegans*

**Figure 5:**
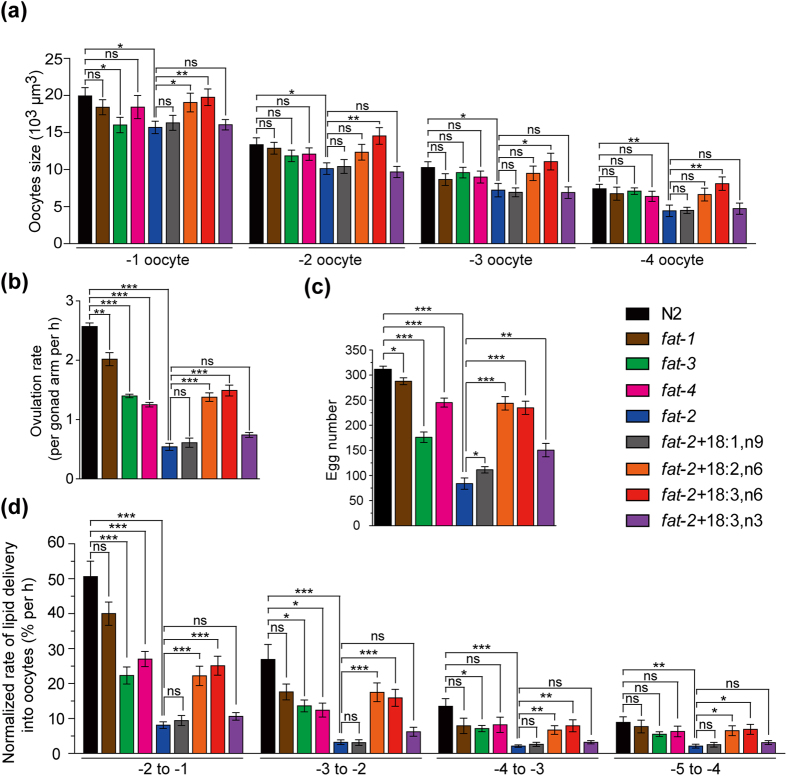
Analyses of oocyte growth, ovulation rate, and egg number of N2 worms and PUFA-deficient mutants. (**a**) The cellular size of the oocytes (n = 9~11), (**b**) ovulation rate (n = 20~32), and **(c)** egg number (n = 10~13) of N2, *fat-1*, *fat-3*, *fat-4*, *fat-2*, and *fat-2* worms supplemented with 18:1,n9, 18:2,n6, 18:3,n6, and 18:3,n3. (**d**) The normalized rate of lipid delivery into oocytes in N2, *fat-1*, *fat-3*, *fat-4*, *fat-2*, and *fat-2* worms supplemented with 18:1,n9, 18:2,n6, 18:3,n6, and 18:3,n3. The data are normalized by wild-type (N2). All worms are 1D-Ad worms. Error bars represent SEM. (P-value: *p < 0.05, **p < 0.01, ***p < 0.001. ns: not significant).

In oocyte development, the size of nearly mature oocytes (the oocytes in the proximal gonad arm) will increase dramatically, which is one of the most important landmark events^24^. We analyzed the cellular volume of the −1 to −4 oocytes in N2, *fat-1*, *fat-3*, *fat-4*, as well as *fat-2* with or without PUFA-supplementation by CARS images (Fig. 5a). The estimated size of the −1 oocyte in N2 worms is ~20000 ± 1100 μm^3^ (Fig. 5a), which is consistent with the volume of fully mature oocytes (21700 ± 4000 μm^3^)^39^. The *fat-1* and *fat-4* oocytes show similar volume, but *fat-2* and *fat-3* oocytes are ~20% smaller compared to the average size of the −1 oocyte in N2 (Fig. 5a). In the PUFA-supplement experiments, we found that omega-6 PUFAs, but not omega-3 PUFAs, recover the oocyte volume of *fat-2* to a level close to N2 (Fig. 5a). The correlation analysis between the oocyte size and lipid uptake suggests that these two parameters are highly correlated (Pearson’s product-moment coefficient r = 0.89) (Fig. S7). It is known that the lipid provision in eggs is important for the development of embryo^26^,^27^. Our data show that *fat-2* mutants have a ~25% lower amount of lipid stored (Fig. 4d) and smaller oocyte size (Fig. 5a) in the −1 oocyte compared to the control, implying that more than a 25% loss of lipid in the oocyte that can cause significantly lethal embryonic defects in *C. elegans*.

We next analyzed the ovulation rate and egg number in N2, *fat-1*, *fat-3*, *fat-4*, and *fat-2* with or without the addition of PUFAs. Compared to N2 worms (~2.5 oocyte per gonad arm per h), we found a slight decrease in *fat-1* worms (~2 oocyte per gonad arm per h), an appreciable decrease in *fat-3* worms (~1.4 oocyte per gonad arm per h) and *fat-4* worms (~1.25 oocyte per gonad arm per h), and a significant decrease in *fat-2* worms (~0.5 oocyte per gonad arm per h) in their ovulation rates (Fig. 5b). Addition of omega-6 PUFAs to *fat-2* worms can partially recover the defect of oocyte ovulation (~1.5 oocyte per gonad arm per h; Fig. 5b), but addition of 18:1,n9 and 18:3,n3 show no significant increase in the ovulation rate of *fat-2* worms. The results of egg number have a similar trend. The egg number (Fig. 5c) has a slight decrease in *fat-1* worms, an appreciable decrease in *fat-3* and *fat-4* worms, and a significant decrease in *fat-2* worms with respect to N2 worms. Addition of omega-6 PUFAs to *fat-2* has a pronounced effect on the recovery of the number of eggs (75% of wild-type).

Oocyte development in *C. elegans* is a continuous process and the reproduction line of oocytes is highly time-dependent. The average transition time from “−n” position to “−(n − 1)” position can be determined as the average time to ovulate a mature oocyte, which is the inverse the ovulation rate. We further calculated the increase of lipid content between −n and −(n − 1) from Fig. 4d. We quantified the rate of lipid delivery into single oocytes as [(the increase of lipid content between adjacent oocytes)/(the average time to ovulate a oocyte)] in wild-type worms, *fat-1*, *fat-3*, *fat-4*, and *fat-2* mutants, as well as PUFA-supplemented *fat-2* worms during oocyte development in live *C. elegans* (Fig. 5d). Compared to the transition of −2 to −1 oocyte in wild-type N2 worms, the lipid delivery rate is slightly lower in *fat-1* (~80%), significantly lower in *fat-3* (~40%) and *fat-4* (~50%), and dramatically lower in *fat-2* (~15%). For the *fat-2* worms with PUFA-supplementation, we found that omega-6 PUFAs, but not omega-3 PUFA and MUFA, restitute the defect in the lipid delivery rate in *fat-2* worms.

**Figure 6:**
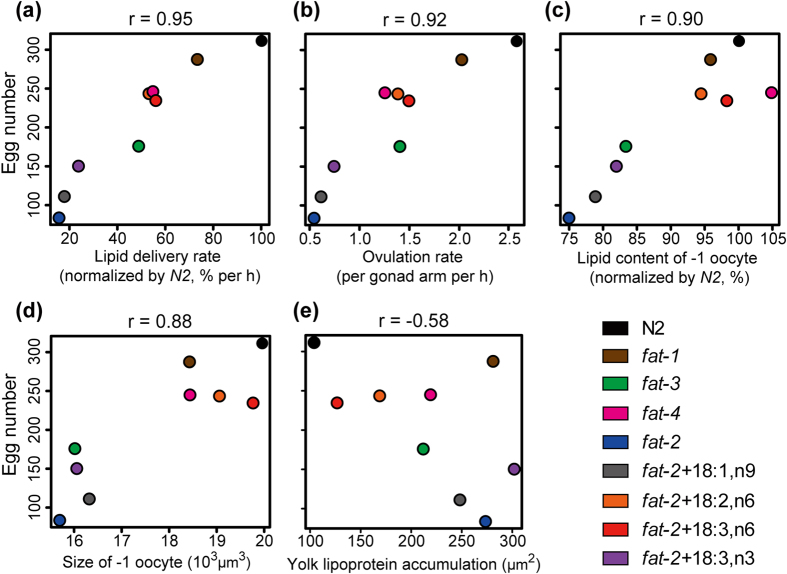
The correlation between various quantitative results and egg number. The scatter plots of (**a**) lipid delivery rate, (**b**) ovulation rate, (**c**) lipid content, (**d**) oocyte size, and **(e)** total area of yolk lipoprotein accumulation *versus* egg number, together with the values of the Pearson’s product-moment correlation coefficient (r). The analyses were conducted using R^40^.

We further analyzed the correlations between the final output results (egg number) and various quantitative measurements by using R^40^. We found that the egg number has highly positive correlations with lipid delivery rate, ovulation rate, lipid content, and oocyte size (r = 0.95, 0.92, 0.90, and 0.88, respectively; Fig. 6a–d), and a moderately negative correlation with the yolk lipoprotein accumulation (r = −0.58; Fig. 6e). The abnormal accumulation of yolk lipoprotein in *fat-1* originates from the over-production of lipid/protein complexes (please see Discussion). If we exclude the data point of *fat-1* in Fig. 6e, then the correlation becomes a strongly negative correlation (r = −0.84), while other correlations are not significantly affected (r = 0.94 for lipid delivery rate; r = 0.91 for ovulation rate; r = 0.92 for lipid content; r = 0.91 for oocyte size). These results suggest that the yolk transportation controlled by omega-6 PUFAs could be one of the important factors that influence the development of oocyte in *C. elegans*.

## Discussion

The *C. elegans* is a good exemplar to verify which kind of PUFA is more critical for the transportation of yolk lipoprotein by 1) designing an input condition (*fat* mutants and the PUFA add-back experiments); 2) quantitatively measuring the parameters involving the development of oocytes (aberrant yolk accumulation, lipid content, lipid delivery rate, oocyte size, and ovulation rate); and 3) eventually collecting the final output results (the reproductive activity, *i.e.* the egg number). We showed that CARS is particularly useful in detecting the aberrant accumulation of yolk lipoprotein, revealing the oocyte lipid content, and estimating the relative rate of lipid delivery into oocytes during oocyte development in live *C. elegans*, demonstrating an alternative label-free method to study the relationship between lipoprotein regulation and oocyte development.

One of the most important issues regarding the study of yolk lipoprotein is to understand the underlying mechanism of how lipids are packaged into the lipid carrier for export. We have demonstrated that the accumulation of secreted yolk lipoprotein in pseudocoelom of live *C. elegans* can be quantitatively measured by CARS microscopy at both protein (~1665 cm^−1^) and lipid (~2845 cm^−1^) Raman bands. However, the measurement of both protein and lipid ratios of intestinal lipid-enriched particles (including mature/immature yolk and lipid storage particles) is not sufficient to identify yolk in the intestine. Yolk lipoproteins are made in the intestine, secreted, and taken up by the developing oocytes. It is believed that lipid molecules translocate between LDs and yolk lipoproteins in the intestine of *C. elegans*^13^. So far, there is no direct evidence to elucidate the interactions between them in the intestine and the underlying mechanism of yolk formation remains unknown. Thus, specific markers or new methods are needed for characterizing those lipid-enriched particles in the intestine.

There are two major yolk lipoprotein complex forms in *C. elegans*, the B dimer and A complex^41^. The B dimer is composed of two yp170B polypeptides, which is encoded by the *vit-2* gene^41^,^42^. The A complex is composed of yp170A, yp115, and yp88. The yp170A is encoded by *vit-5*, while yp115 and yp88 are the two smaller yolk lipoproteins cleaved from a precursor protein encoded by *vit-6*^41^,^42^. The exogenous *vit-2::gfp* is widely used as a fluorescent marker to indicate the distribution of GFP-tagged B dimer yolk lipoprotein complex (yp170B). To quantify the amount of delivered lipid in oocytes, the contribution of other lipid delivery pathways should be considered. In this work, we compared the integrated GFP signal and integrated CARS signal collected from the oocytes of the wild-type worms with *vit-2p::vit-2::gfp* transgene expression, and discovered a strong linear correlation between them (R^2^ > 0.99). The interception of Fig. 4b is not zero at the axis of the Δ integrated CARS signal, which could be due to the contribution of the A complex, the endogenous B dimer, or an unknown lipid delivery pathway that cannot be detected by the fluorescent signal of GFP-tagged yp170B. Since the expression of GFP-tagged yolk lipoprotein may not accurately reflect the amount of total yolk lipoprotein, along with some possible perturbation from exogenous protein expression, the proposed label-free CARS imaging method is more reliable for quantifying and comparing the uptake of yolk lipoprotein/oocyte lipid content among different strains.

The normalized rate of lipid delivery in oocyte transitions in live *C. elegans* is established for the first time. We discovered a 50% lipid increase during the transition of the −2 to −1 oocyte in all of our experimental strains. This result is consistent with the observation of the GFP signal in the N2 worms expressing VIT-2-GFP, indicating that the major target of yolk lipoprotein complexes is the near mature oocytes (−2 and −1 oocytes). Both *fat-3* and *fat-4* mutants show decreased lipid delivery rates from −5 to −1 oocytes (49% and 54% of the N2 worms, respectively). The *fat-2* mutant exhibits a dramatic decrease in the lipid delivery rate (only ~16% of the control). Although *fat-1* mutant shows slightly lower oocyte lipid content and delivery rate than the control, the oocyte growth and reproduction of *fat-1* mutant are not affected. The rescue experiments show that omega-6 PUFAs, but not omega-3 PUFAs, can increase the lipid delivery rate in *fat-2* mutants. These results suggest that due to the lack of omega-6 PUFAs, the development of oocytes takes a much longer time (e.g. low ovulation rate) to acquire sufficient nutrients for embryonic development. The delivery of lipid into oocytes can be more efficient with sufficient omega-6 PUFAs.

The roles of omega-3 and -6 PUFAs in oocytes and embryo development are still in debate. It is not clear why the *fat-1* mutant has non-detectable omega-3 PUFAs and increased levels of omega-6 PUFAs (about 13-fold higher arachidonic acid levels than wild-type worms) but shows no apparent defects in reproduction^18^,^43^, while *fat-2* and *fat-3* mutant shows reduced brood size and live progeny^17^,^44^. We noticed that *fat-1* mutant shows a high level of secreted yolk lipoprotein accumulation, but the oocyte lipid content in the *fat-1* mutant is similar to the level of wild-type N2 worms. It is found that the overexpression of PUFAs can negatively regulate DAF-16/FOXO to promote the protein expression level of yolk lipid protein (vitellogenins) in the intestine^23^. Based on our findings, we suggest that the over-production of omega-6 PUFAs in *fat-1* mutant results in the generation of abundant yolk lipoprotein, which exceeds the uptake limit of oocytes. As a result, the *fat-1* mutant shows abnormal yolk lipoprotein accumulation in pseudocoelom but no apparent defect in reproduction, suggesting that omega-3 PUFAs are not critical for the reproductive process. Edmonds *et al*. reported that supplementation with arachidonic acid (20:4,n6) reduces the accumulation of yolk lipoprotein in pseudocoelom of *fat-2(wa17); vit-2p::vit-2::gfp* transgenic worms^21^. Watts *et al*. found that biochemical complementation with the omega-6 PUFA (18:3,n6) restores the reproductive defect in the *fat-3* mutant^44^. Together with our results from PUFA-supplementation experiments, all evidence strongly supports that omega-6 PUFAs, but not omega-3 PUFAs, are required for yolk lipoprotein transportation and oocyte development in *C. elegans*.

It is found that Omega-6 PUFAs such as linoleic (18:2,n6) and arachidonic (20:4,n6) acids are considerably enriched in the oocytes of cow, sheep and pigs^1^. In human oocytes, the most abundant fatty acids are saturated fatty acids (~79% of total fatty acids) followed by MUFA (~14%) and omega-6 PUFAs (~5%) from fertilization-failed human oocytes^45^. These data indicate that omega-6 PUFAs are associated with the development of oocytes in mammals. The results of *in vitro* incubation of bovine oocytes with omega-6 PUFA show that linoleic acid (18:2,n6) can stimulate lipid accumulation in maturing bovine oocytes in a dose dependent manner (in the 0~50 μM range)^31^. Microarray analysis reveals that the expression of the LDL receptor increases more than 10-fold during the maturation of human oocytes^46^,^47^. Since both yolk lipoprotein receptor (RME-2) and human LDL receptor are in the LDL receptor family^16^, it is possible that the regulatory effect of omega-6 PUFAs found in this study may similarly influence the endocytosis of lipoprotein in mammals.

In conclusion, we established an experimental method to quantitatively measure the level of aberrant yolk lipoprotein accumulation and lipid delivery into oocytes in living *C. elegans* without any labeling. We also demonstrated the first systematic examination of yolk lipoprotein transportation, lipid delivery, and oocyte development in wide-type, PUFA-deficient mutants, and various PUFA-supplemented *fat-2* worms. Together with the results of egg number, we conclude that the omega-6 PUFAs is an important factor that modulates the delivery of lipid and further regulates the development of oocyte in *C. elegans*. This work remarks the value of using CARS as a molecular-selective label-free imaging technique for the study of yolk lipoprotein, PUFA regulation, and oocyte development in *C. elegans*, with implications for application to developmental biology and reproductive biology.

## Methods

### Worms and reagents

Wild-type (Bristol N2), DH1390 [*rme-2*(b1008)], BX24 [*fat-1*(wa9)], BX26 [*fat-2*(wa17)], BX30 [*fat-3*(wa22)], and BX17 [*fat-4*(wa14)] mutant *C. elegans* were obtained from the Caenorhabditis Genetics Center. All strains used in this study were cultured on nematode growth medium (NGM) plates seeded with *E. coli* OP50 and incubated at 20 °C. The late L4 larvae were picked onto plates and incubated at 20 °C to mature into young adults. After that, the worms were incubated another 24 h for them to grow into one-day-adult (1D-Ad) worms. Levamisole was purchased from Sigma (St. Louis, Missouri) and used to paralyze worms for microscopy.

### Dietary supplementation of fatty acids in worms

Fatty-acid-containing NGM plates were prepared as previously described^48^. Briefly, fatty acids were added slowly to the cooled NGM media to a final concentration of ~0.2 mM. Plates were seeded with OP50 bacteria and kept in the dark overnight. OP50 bacteria accumulated PUFAs into their lipids at ranges from 1–5%^48^.

### Raman micro-spectroscopy

Raman measurements of secreted yolk lipoprotein accumulation in *C. elegans* were performed by a Raman microscope (HR800, Horiba Scientific, Kyoto, Japan). A HeNe laser beam (632.8 nm) was focused on the sample through a 50× objective. The Raman scattering was collected by the same objective and delivered to an 80-cm spectrograph equipped with a liquid nitrogen-cooled CCD for spectral analysis. Raman spectra were collected in the region of 900–1,800 cm^−1^ with ∼60 s acquisition time.

### Analysis of secreted yolk lipoprotein accumulation in the pseudocoelomic cavity of uterus

The following is the process for quantifying yolk lipoprotein accumulation in the pseudocoelomic cavity of uterus (here we used 1D-Ad N2 worms as an example): First, the measured sizes of yolk lipoprotein accumulations from all worms were pooled together to plot the size histogram. Figure S6a shows the size histogram of yolk lipoprotein accumulations from total 57 1D-Ad N2 worms. Second, the number of occurrence per binned area size (0~20 μm^2^, 20~40 μm^2^, 40~60 μm^2^, etc.) was divided by the number of worms (n = 57) to yield the distribution of the average frequency to find a corresponding size of accumulation in a worm (Fig. S6b). Finally, the area contribution histogram (Fig. S6c) was obtained by multiplying the frequency in Fig. S6b by each binned area size. The area contribution curve (red line in Fig. S6c) provides the contribution of each accumulation size in the pseudocoelomic cavity of uterus in a worm. The integration of the whole area contribution curve gives the total area of yolk lipoprotein accumulations in the pseudocoelomic cavity of uterus in a worm.

### CARS/TPEF microscopy

Figure S8 describes the setup of the CARS microscope. The acquisition times were ∼5 s for a CARS image and ∼2 s for a two-photon excitation fluorescence (TPEF) image. CARS or TPE-F image was obtained within an appropriate field of view (∼141 × 141 μm^2^) allowing minimal non-uniformity of illumination. To preserve the largest dynamic range for further CARS image analysis, the data were recorded as 12-bit gray-level images. All images were analyzed by the ImageJ software^49^. The CARS spectrum was analyzed from the CARS images with vibrational frequencies in the range of 2,800–3,050 cm^−1^.

### Quantitative analysis of CARS/TPEF signal in individual oocytes

The quantitative analysis of lipid content in oocytes was modified from our previous image analysis method^50^,^51^. CARS images of oocytes were taken at 2845 cm^−1^ and analyzed by image J software. Background intensity, which was mainly contributed from the non-resonant signal of non-lipid-rich cellular structures, was determined and subtracted from the average CARS intensity of the oocyte nucleus. The integrated CARS intensity was calculated by summing the square roots of the background-subtracted CARS intensities of the pixels that belongs to the oocyte (Fig. S9), which yields a numerical value for the lipid content in the single oocyte. For the integrated GFP intensity, the background intensity was defined as the average fluorescence intensity in the VIT-2::GFP negative region for which the definition was described by Yi *et al*.^19^. The integrated GFP intensity was then determined by the summation of the background-subtracted GFP intensities of the pixels that belongs to the oocyte.

### Estimation of oocyte size

The single oocyte cell was assumed as a cylinder^24^, so its volume =  π × length × (width/2)^2^. Area and length measurements of oocytes were analyzed with ImageJ software.

### Egg number and oocyte ovulation assay

The egg number was used to quantify the number of eggs laid over the worm’s reproductive lifetime. The egg number of each worm was defined as the sum of laid eggs (including non-hatched and hatched eggs). The procedure of the oocyte ovulation assay was described by Brisbin *et al*.^52^. The number of oocytes/embryos in 1D-Ad worms was counted by the Nikon SMZ645 microscope. Then, the 1D-Ad worms were picked into a new plate and incubated for 5–8 h at 20 °C to lay eggs. The number of eggs laid on the plate as well as the number of eggs retained in worms were then counted. Ovulation rate, the average number of oocytes ovulated in 1 h per gonad arm, was determined by the following calculation: (the number of eggs laid on the plate + the number of oocytes/embryos retained in worms) − (the number of oocytes/embryos originally in the worms), and then averaged for the time period (5–8 h) as well as the number of gonad arms.

## Additional Information

**How to cite this article**: Chen, W.-W. *et al*. Specific polyunsaturated fatty acids modulate lipid delivery and oocyte development in *C. elegans* revealed by molecular-selective label-free imaging. *Sci. Rep.* **6**, 32021; doi: 10.1038/srep32021 (2016).

## Supplementary Material

Supplementary Information
