# Supplementary material for: Specific polyunsaturated fatty acids modulate lipid delivery and oocyte development in C. elegans revealed by molecular-selective label-free imaging
Source: Sci Rep. 2016 Aug 18;6:32021. doi: 10.1038/srep32021 (PMC4989181; doi:10.1038/srep32021)
Supplement: Supplementary Information [file srep32021-s1.pdf]

Specific polyunsaturated fatty acids modulate lipid delivery and oocyte development in *C. elegans* revealed by molecular-selective label-free imaging

Wei-Wen Chen[1,2,3,+], Yung-Hsiang Yi[4,5,+], Cheng-Hao Chien[1], Kuei-Ching Hsiung[5], Tian-Hsiang Ma[5], Yi-Chun Lin[1], Szecheng J. Lo[4,5,*], and Ta-Chau Chang[1,2,*]

[1]Institute of Atomic and Molecular Sciences, Academia Sinica, Taipei 106, Taiwan
[2]Molecular Science and Technology Program, Taiwan International Graduate Program, Academia Sinica, Taipei 106, Taiwan
[3]Department of Chemistry, National Tsing Hua University, Hsinchu 300, Taiwan
[4]Center of Molecular Medicine and [5]Department of Biomedical Sciences, College of Medicine, Chang Gung University, Kwei-Shan, Tao-Yuan 333, Taiwan

+these authors contributed equally to this work

* corresponding author

Prof. Szecheng J. Lo	email: losj@mail.cgu.edu.tw
Tel: 886-3-211-8800 ext.3405	Fax: 886-3-211-8700
Prof. Ta-Chau Chang	email: tcchang@po.iams.sinica.edu.tw
Tel: 886-2-2366-8231	Fax: 886-2-23620200

SUPPLEMENTARY INFORMATION

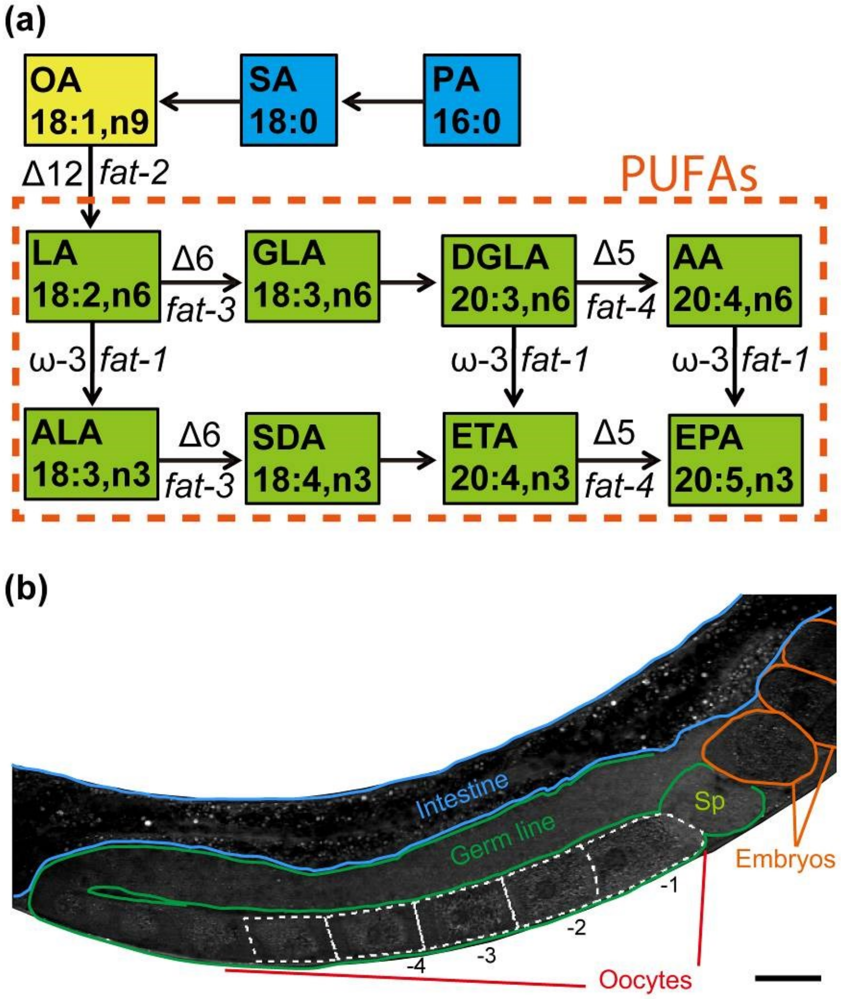

Figure S1. Lipid synthesis and distribution in *C. elegans*. **(a)** Lipid synthesis pathway in *C. elegans*. PA: palmicitc acid; SA: stearic acid; OA: oleic acid; LA: linoleic acid; GLA: gamma-linolenic acid; DGLA: Dihomo-gamma-linolenic acid; AA: arachidonic acid; ALA: alpha-linolenic acid; SDA: stearidonic acid; ETA: eicosatetraenoic acid; EPA: eicosapentaenoic acid. Δ12 for delta-12 desaturase (encoded with *fat-2* gene); ω-3 for omega-3 desaturase (encoded with *fat-1* gene); Δ6 for delta-6 desaturase (encoded with *fat-3* gene); Δ5 for delta-5 desaturase (encoded with *fat-4* gene). The *fat-1* gene encoding enzyme, omega-3 desaturase, can convert all omega-6 PUFAs into omega-3 PUFAs.[1] The *fat-2* gene encoding enzyme, delta-12 desaturase, plays a critical role in converting the monounsaturated fatty acid (MUFA) into various PUFAs.[1] The other two enzymes, delta-6 desaturase (encoded by *fat-3* gene) and delta-5 desaturase (encoded by *fat-4* gene), are involved in the biosynthesis of C20 PUFAs.[1] **(b)** The CARS image (at 2845 cm^{-1}) of a partial wild-type N2 worm shows the intestine and gonad. Sp stands for spermatheca. The dashed line highlights individual oocytes, and the numbers indicate the positions of each oocytes; the nearest one to Sp is designated as -1. Scale bar = 30 μm.

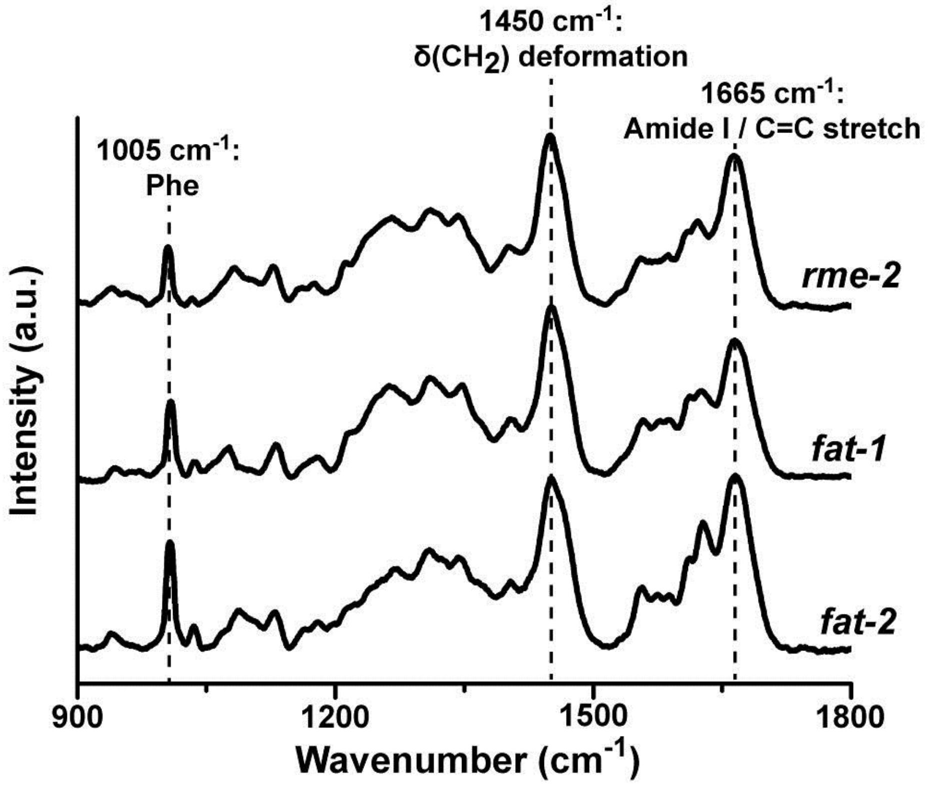

Figure S2. Raman spectra of yolk lipoprotein accumulation in *rme-2*, *fat-1*, and *fat-2* mutants. Raman peak assignment: 1003 cm^{-1} for phenylalanine (Phe),[2] 1450 cm^{-1} for δ(CH2) deformation vibration,[3] and 1665 cm^{-1} for amide I / C=C stretch.[2]

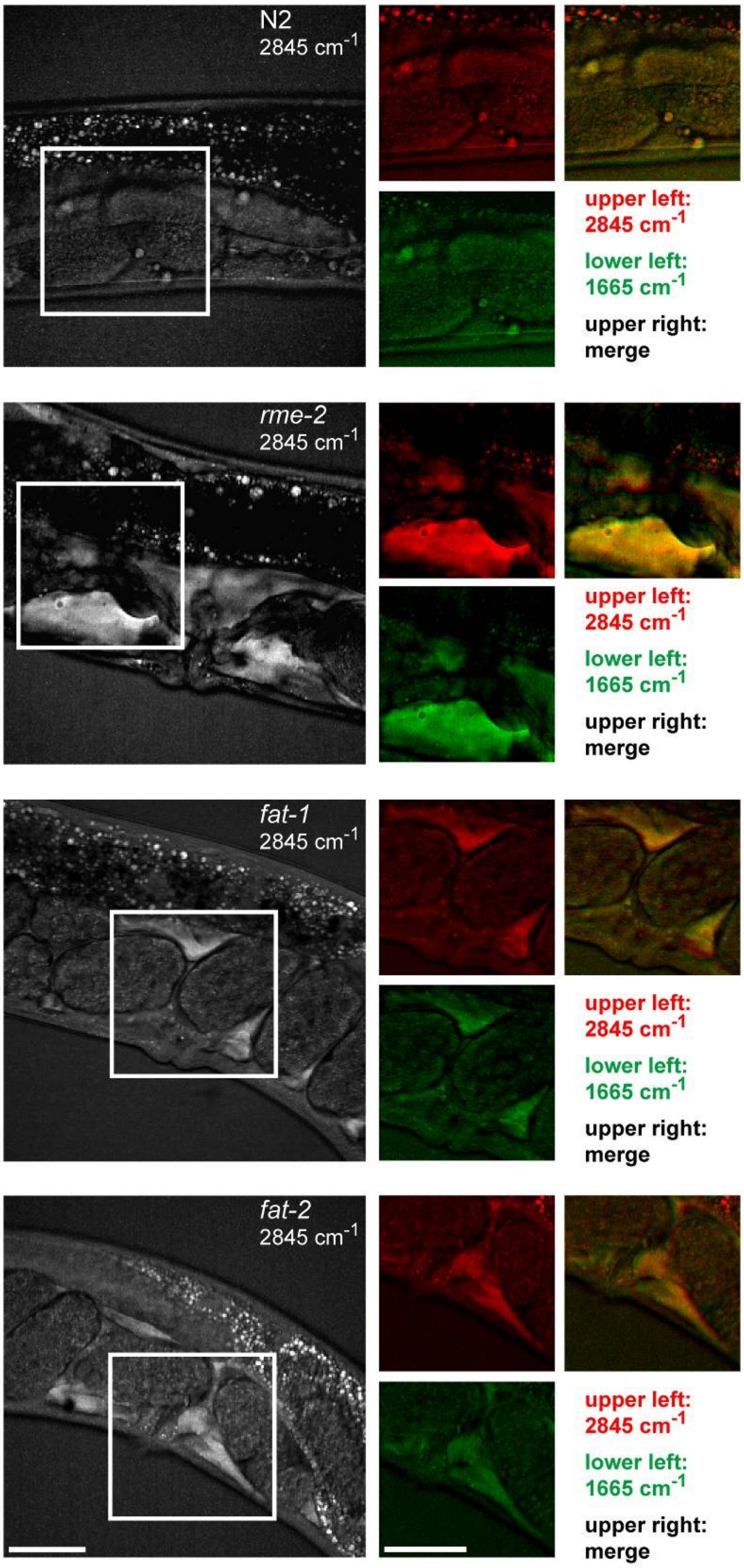

Figure S3. CARS imaging of *rme-2*, *fat-1*, and *fat-2* mutants at ~2845 cm^{-1} (lipid band, in red) and ~1665 cm^{-1} (protein band, in green). The merged images showed highly co-localization of yolk lipoprotein accumulation in CARS lipid and CARS protein images. Scale bar = 30 μm.

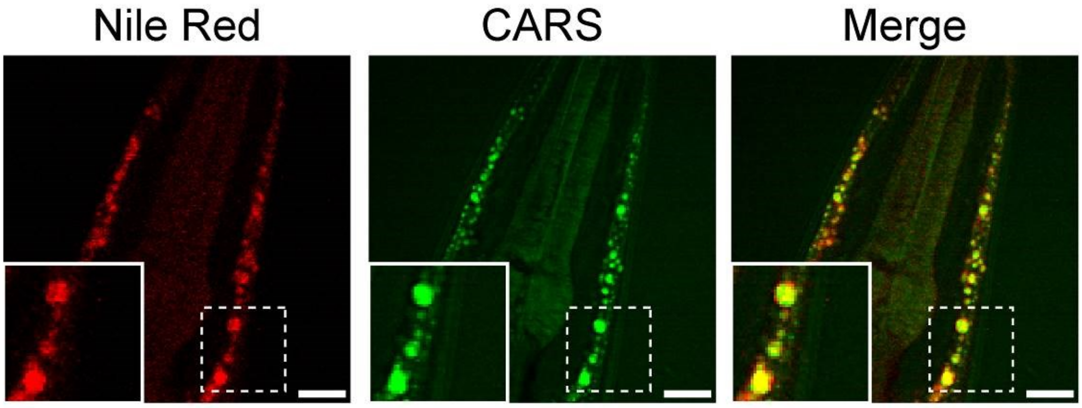

Figure S4. The comparison of Nile Red (red) and CARS (at 2845 cm^{-1}, green) signals in detecting lipid droplets in the skin-like hypodermal cells of one-day adult (1D-Ad) wild-type worms. The fixed Nile Red staining protocol was adopted from the previous reported method.[4] Scale bar = 10 μm.

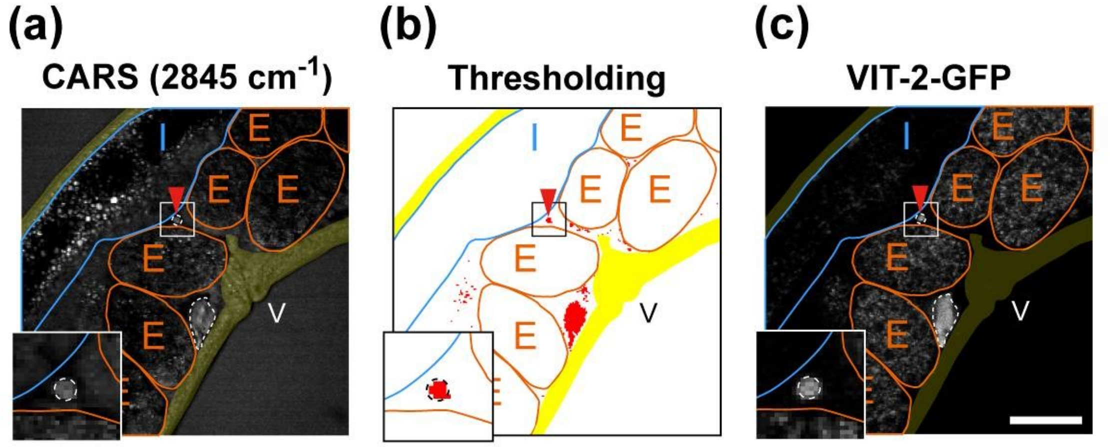

Figure S5. CARS and TPEF imaging of a 1D-Ad wild-type worm. **(a)** The CARS image, **(b)** the binary image (red and white) after applying the thresholding method, and **(c)** the VIT-2-GFP fluorescence image of the same worm. Red arrows indicate the same yolk lipoprotein accumulation, whose size is ~ 9.5μm^2. Scale bar = 30 μm.

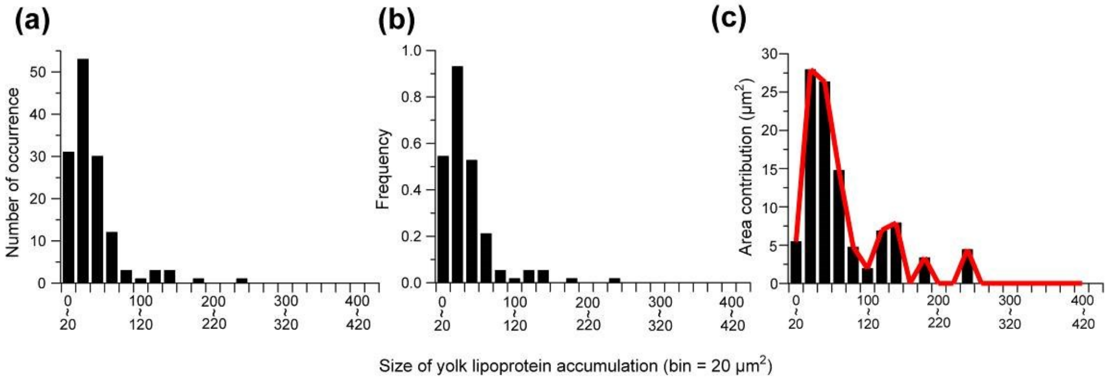

Figure S6. The analysis of yolk lipoprotein accumulation in the pseudocoelomic cavity of uterus in 1D-Ad wild-type worms (total 57 worms). **(a)** The size histogram of yolk lipoprotein accumulation. **(b)** The distribution of the average frequency to find a corresponding size of accumulation in a worm. **(c)** The area contribution histogram, obtained by multiplying the frequency by each binned area size. The red line stands for the area contribution curve.

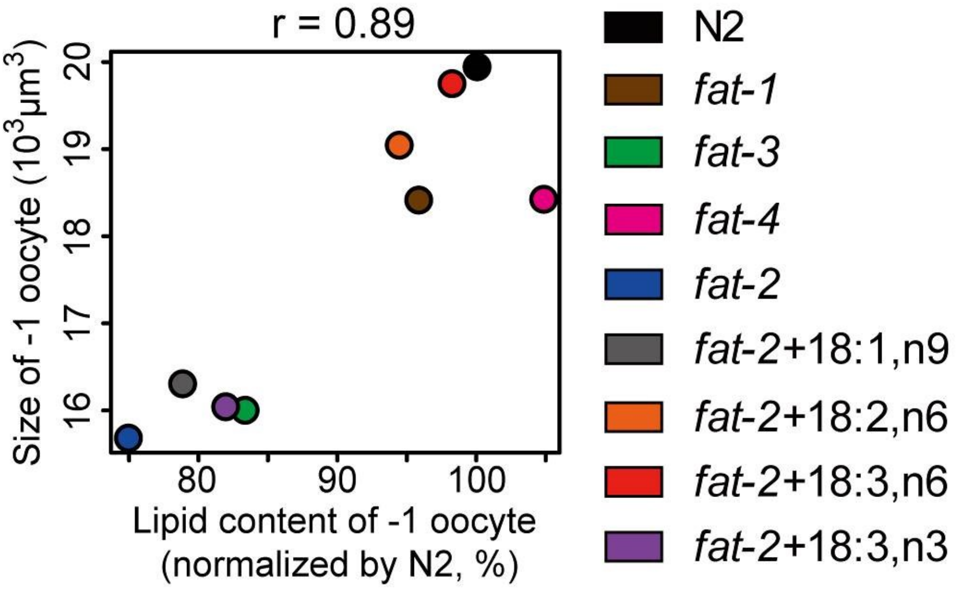

Figure S7. The correlation between lipid content and oocyte size together with the analyzed result of the Pearson's product-moment correlation coefficient (r=0.89).

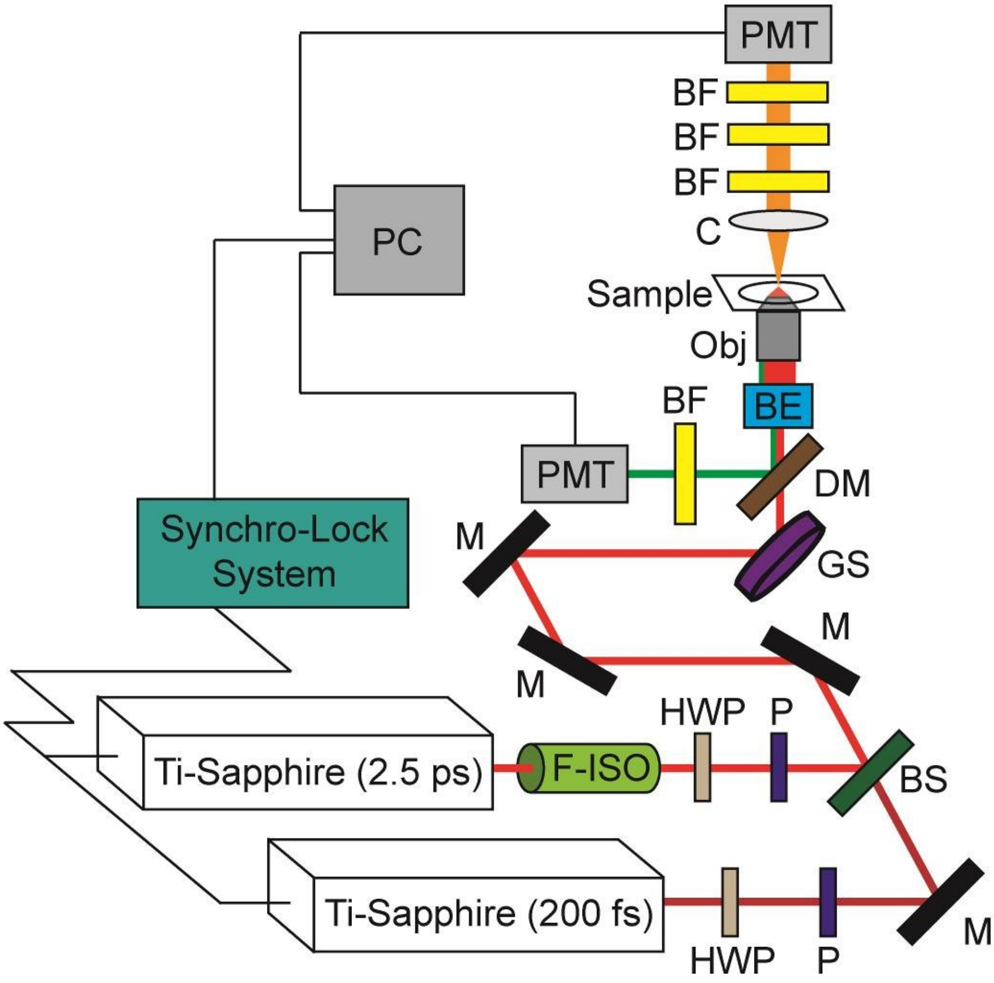

Figure S8. The setup of CARS microscopy. Briefly, two Ti-sapphire laser beams (Mira-900F as the Stokes beam and Mira-900P fixed at ~710nm as the pump/probe beam, Coherent, California) were synchronized by Synchro-Lock system (Coherent, California). The vibrational frequency difference between two laser beams was adjusted to match lipid band (~2845 cm^{-1}) or protein band (~1665 cm^{-1}) for CARS imaging. The two laser beams were collinearly combined and guide into a laser scanning microscope (FV300 and IX-71, Olympus, Tokyo, Japan), and focused onto the sample by a 40X N.A. = 0.9 objective (UPLSAPO 40X, Olympus, Tokyo, Japan). The forward CARS signal was collected by a condenser (N.A. = 0.55), passing through three band-pass filters (FF01-630/92*2 and FF-01590/10*1, Semrock, New York), and detected by a photomultiplier tube (PMT) (R7400U-02, Hamamatsu, Japan). Fluorescence signal of GFP was collected by the same 40X objective, passing through a band-pass filter (FF01-512/25, Semrock, Rochester, New York), and detected by a photomultiplier tube (PMT , R3896, Hamamatsu, Japan). F-ISO: Faraday isolator. BS: beam splitter. HWP: half-wave plate. P: polarizer. M: mirror. GS: galvo scanner. DM: dichroic mirror. BF: band-pass filter. C: condenser. BE: beam expander. Obj: objective. PMT: photomultiplier tube.

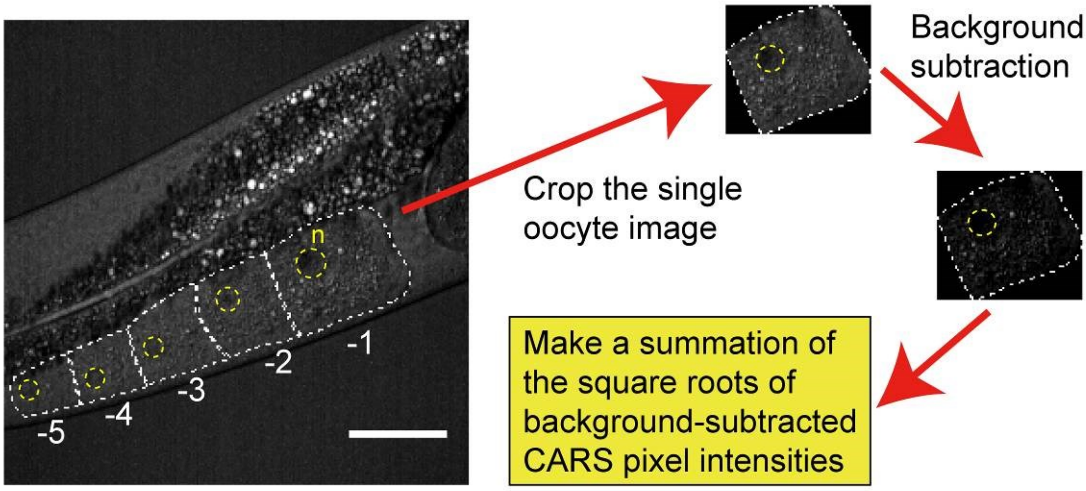

Figure S9. Steps of quantitative analysis of lipid storage in single oocytes. The single oocyte image was first cropped. Then, the background noise was determined by the average intensity of non-lipid-rich region, *e.g.* nucleus (indicated by yellow circle). Finally, the square root values of background-subtracted pixel intensities were integrated. For each worm, the first 5 oocytes (-1 to -5 oocytes) were quantitatively analyzed. Scale bar = 30 μm.

Reference

1 Watts, J. L. & Browse, J. Genetic dissection of polyunsaturated fatty acid synthesis in Caenorhabditis elegans. *P. Natl. Acad. Sci. U.S.A.* **99**, 5854-5859 (2002).

2 Camp, C. H. *et al.* High-speed coherent Raman fingerprint imaging of biological tissues. *Nat. Photonics* **8**, 627-634 (2014).

3 Le, T. T., Duren, H. M., Slipchenko, M. N., Hu, C. D. & Cheng, J. X. Label-free quantitative analysis of lipid metabolism in living Caenorhabditis elegans. *J. Lipid Res.* **51**, 672-677 (2010).

4 Yi, Y. H. *et al.* Lipid droplet pattern and nondroplet-like structure in two fat mutants of Caenorhabditis elegans revealed by coherent anti-Stokes Raman scattering microscopy. *J. Biomed. Opt.* **19**, 011011 (2014).
